# The use of medication and nutritional supplements during FIFA World Cups 2002 and 2006

**DOI:** 10.1136/bjsm.2007.045187

**Published:** 2008-03-04

**Authors:** P Tscholl, A Junge, J Dvorak

**Affiliations:** FIFA Medical Assessment and Research Center (F-MARC), Schulthess Klinik, Zurich, Switzerland

## Abstract

**Objective:**

To examine medication use in male top-level football players prior to and during international tournaments.

**Design:**

Prospective survey.

**Material:**

2944 team physicians’ reports on players’ medication intake.

**Methods:**

Each team physician was asked to document all medication and nutritional supplements taken in the 72 h prior to each match.

**Results:**

A total of 10 384 substances were reported (1.8 substances/player/match); 4450 (42.9%) of these were medicinal and 5934 (57.1%) nutritional supplements. The medications prescribed most frequently were non-steroidal anti-inflammatory agents (n = 2092; 20.1%); more than half of the players took these at least once during a tournament and more than 10% prior to every match (156 out of 1472). β-2-Agonists were reported for 1.4% (n = 20) and inhaled corticosteroids for 1.6% (n = 23) of participating players. Injected corticosteroids were reported for 73 players.

**Conclusions:**

The high intake of medication in international football – especially of non-steroidal anti-inflammatory drugs – is alarming and should be addressed. The results raise questions as to whether the medication was taken solely for therapeutic reasons. In view of the potential side effects, more restrictive recommendations for sport need to be developed.

The legal and illegal use of medical substances is widespread in international sport and is growing rapidly in its complexity.^1^ Most current literature focuses on illegal substances such as anabolic steroids,^1^ growth hormone,^2^ erythropoietin and blood doping.^3^ Although many authors have raised concerns about the use in international sports of prescribed substances such as β2-agonists,^4^ non-steroidal anti-inflammatory drugs (NSAIDs),^5^^–^7 corticosteroids,^8^ and nutritional supplements,^9^ ^10^ only little is known about the magnitude of their current use.

The main principles in sport concern not only equality and fairness but also health.^10^ ^11^ As such, the responsibilities of sports associations include not only the establishment of doping control networks but also the investigation of legally prescribed, but potentially harmful medication – and not only its use in excess.

Previous studies of athletes participating in the Olympic Games 2000 in Sydney found that 80% of athletes declared using some sort of medication.^10^ A mean intake of 4.6 dietary supplements per player, prescribed medications and over-the-counter substances were reported for Canadian athletes.^9^

Recently published data on medication use in professional footballers indicate a high intake of both supplements^12^ and non-steroidal anti-inflammatory drugs.^12^ ^13^ However, little scientific evidence exists regarding the beneficial effects of nutritional supplements on sporting performance.^14^

The Fédération Internationale de Football Association (FIFA) adopted antidoping measures in 1970, and since then has continuously developed its strategy.^1^ The low incidence of positive doping samples in professional football (0.4%) is testament to the efforts made by FIFA to contain the problem over the last 40 years.^1^ Just before the FIFA World Cup 1998 in France, FIFA initiated a new approach, to record the use of medication and nutritional supplements in each player prior to each match of a major tournament; to the authors’ knowledge, FIFA is the first international sports ruling body to introduce such an initiative.

## METHODS

### Data collection

In connection with the doping controls carried out in the FIFA World Cup tournaments 2002 and 2006, all team physicians were asked to enter in English in legible handwriting “any medication taken by the players or administered to them in the 72 hours preceding the match… The team doctor shall also note down any non-prescription medicines or food supplements taken by the players”.^15^

### Classification

Based on the information provided by the team physicians, the active pharmaceutical ingredient of each reported substance was identified, and the substance was classified as one of the following:

- Painkilling and anti-inflammatory drugs
-
  - NSAIDs.^5^ ^16^
  - Analgesics.
  - Injected corticosteroids and local anaesthetics.
- Muscle relaxants.
- Respiratory agents – anti-asthmatic, antihistaminic, sympathomimetic medication, and others.
- Medication for intestinal purposes – proton pump inhibitors, corticosteroids, and others.
- Antimicrobial medication.
- Others – psychotropic substances, topical corticosteroids and others.
- Supplements – macronutrients and micronutrients, herbal supplements^9^ (derivatives from plant sources), and others.

### Participating players

Thirty-two countries (23 players each) qualified for each of the FIFA World Cup tournaments. A total of 64 matches took place from the qualification round up to the World Cup final, with three being the lowest number of matches played by any team for one tournament, and seven being the highest number. Each World Cup included 2944 player matches.

### Data presentation

The incidence of substance intake was calculated as follows:

- substance/player/match^10^ (mean intake per player)
- number of individual players reported to be using a substance^10^
- substance/player/tournament^9^

### Data analysis

The statistical methods applied were frequencies, cross-tabulations and Pearson’s correlation.

We used χ^2^ tests for comparison of substance categories. 95% confidence intervals were calculated according the following formula: 95% CI =  incidence ±1.96× (incidence/square root (number of incidents)). Statistical significance was accepted at p<0.05 in all cases.

## RESULTS

**Figure 1:**
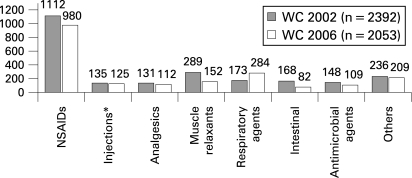
Prescribed medication during FIFA World Cup 2002 and 2006. *Corticosteroid and local anaesthetic injections only.

In the two FIFA World Cups, a total intake of 10 384 substances was documented, of which more than half (57.2%) were classified as nutritional supplements. The average consumption per player was 1.8 substances (0.8 medications) prior to each match (see fig 1). Some individual players took as many as seven different substances or five of the seven different substance groups prior to a match.

During the two tournaments, 44.0% of the players took at least one type of medication prior to a match, and 68.5% during the tournament; 19.7% of players had no medication intake (see table 1).

**Table 1:** Number of players with reported medication and use of nutritional supplements

	WC 2002		WC 2006	
	No. of players		No. of players	
	Per match	During tournament	Per match	During tournament
	(n = 2944) (%)	(n = 736) (%)	(n = 2944) (%)	(n = 736) (%)
Any medication	1335 (45.3)	500 (67.9)	1257 (42.7)	508 (69.0)
NSAIDs	960 (32.6)	403 (54.8)	855 (29.0)	399 (54.2)
Injections*	120 (4.1)	77 (10.5)	103 (3.5)	58 (7.9)
Analgesics	131 (4.4)	91 (12.4)	108 (3.7)	83 (11.3)
β-2-Agonists	34 (1.2)	8 (1.1)	31 (1.1)	12 (1.6)
Antihistamines	60 (2.0)	43 (5.8)	106 (3.6)	55 (7.5)
Any supplement	925 (31.4)	314 (42.7)	1041 (35.4)	317 (43.1)
Any substance	1809 (61.4)	582 (79.1)	1868 (63.5)	600 (81.5)

*Corticosteroid and local anaesthetic injections only.

### Non-steroidal anti-inflammatory drugs and analgesics

NSAIDs were the most frequently prescribed substances, accounting for almost half of all reported medicines used (2002: 46.5%, 2006: 47.7%). More than half of the players took NSAIDs at least once during a tournament and 30.8% prior to a match. On average, 22.9% of the players (2002: n = 183; 2006: n = 154) were using NSAIDs in two out of three matches, and 10.6% of the players (2002: n = 80; 2006: n = 76) were taking them prior to every match. Substantial differences were observed between the countries; in one team 22 out of 23 players took NSAIDs prior to every match (for details, see table 2).

**Table 2:** Reported medicaments and supplements per match in relation to the participating countries (average intake of one substance per match)

Team	NSAIDs		Injected corticosteroids		Injected LA		Analgesics		β2-Agonists		Antihistamines		Inhaled corticosteroids		Supplements		Same doctor
	WC 2002/2006		WC 2002/2006		WC 2002/2006		WC 2002/2006		WC 2002/2006		WC 2002/2006		WC 2002/2006		WC 2002/2006		*yes/no*
A																	
1	21.4	14.7	2.0	0.3	0.0	0.0	2.4	0.3	0.8	2.0	0.2	2.7	0.0	1.0	0.0	1.7	y
2	9.9	7.7	0.6	0.7	5.1	1.9	0.6	0.6	1.0	0.0	0.0	2.7	0.0	1.1	6.1	1.7	y
3	11.3	8.6	0.0	0.1	0.0	0.1	0.0	3.3	0.0	0.4	0.0	0.1	0.0	0.4	135.0	109.6	y
4	13.3	17.3	0.0	1.7	0.3	1.0	8.3	12.7	0.0	0.0	4.0	9.7	0.0	0.0	25.7	45.7	y
5	7.9	10.3	0.6	0.7	0.4	0.0	3.3	0.3	0.0	0.0	3.3	0.3	0.0	0.3	0.0	0.0	y
6	8.7	7.2	3.7	0.4	0.3	0.6	0.0	0.0	0.0	0.0	0.0	0.4	0.0	0.0	37.7	46.0	y
7	3.5	6.0	0.0	0.3	0.3	0.3	0.3	0.5	2.8	2.3	1.8	0.0	1.0	1.0	0.0	2.0	y
8	4.8	3.3	0.5	0.3	1.0	2.0	0.0	0.0	0.8	0.0	0.3	0.0	0.0	0.0	1.0	0.0	y
9	37.3	12.7	0.3	0.0	0.0	4.7	5.8	0.3	0.0	0.0	0.5	0.0	0.0	0.3	46.0	162.3	n
10	0.5	33.3	0.0	0.0	0.0	0.0	0.0	0.8	0.0	0.0	0.0	0.0	0.0	0.0	65.0	62.5	n
11	7.6	7.2	0.0	0.0	0.0	0.0	2.0	1.0	0.0	0.0	0.1	0.8	0.0	0.0	0.9	1.8	n
12	4.8	10.0	0.0	0.0	0.0	0.0	0.4	0.4	1.0	0.0	0.0	1.0	0.0	0.2	0.0	0.0	n
13	10.7	13.3	0.0	0.3	2.3	3.7	1.0	1.0	0.0	0.0	0.3	3.0	0.0	0.0	1.0	0.3	n
14	10.5	1.9	0.3	1.6	1.3	1.3	0.0	0.0	0.0	0.0	0.0	0.6	0.0	0.3	0.0	0.0	n
15	5.0	4.5	0.8	0.0	0.2	0.0	0.4	0.0	0.0	0.0	0.0	0.3	0.0	0.0	62.2	51.5	n
16	3.0	4.3	0.0	0.0	0.0	0.1	0.0	1.3	0.0	0.6	0.0	0.3	0.0	0.4	0.0	0.0	n
17	3.0	4.3	0.0	0.0	0.0	0.0	0.0	0.3	0.0	0.0	0.0	0.0	0.0	0.0	30.7	32.3	n
18	3.3	4.0	0.0	0.0	0.0	0.0	0.0	0.0	0.0	0.0	0.0	0.0	0.0	0.0	0.0	0.0	n
19	5.0	1.7	0.0	0.0	0.0	0.0	0.0	0.0	0.0	0.0	0.0	0.0	0.0	0.0	0.0	94.7	n
20	2.3	4.0	0.0	1.0	0.7	0.0	0.7	0.3	0.0	0.0	1.7	0.7	0.0	0.0	4.0	208.3	n
B																	
1	12.4	–	0.1	–	0.3	–	0.9	–	0.0	–	1.7	–	0.0		19.7	–	
2	15.0	–	0.7	–	1.3	–	0.3	–	0.0	–	0.0	–	0.0		24.3	–	
3	14.7	–	0.7	–	0.0	–	0.0	–	0.0	–	0.0	–	0.0		51.0	–	
4	10.3	–	0.0	–	0.0	–	0.5	–	1.0	–	0.0	–	0.0		0.3	–	
5	9.8	–	0.0	–	0.0	–	0.8	–	0.0	–	0.0	–	0.0		0.5	–	
6	5.2	–	0.0	–	0.0	–	0.8	–	0.0	–	0.0	–	0.0		0.4	–	
7	7.3	–	0.0	–	0.0	–	0.0	–	0.0	–	0.0	–	0.0		0.3	–	
8	6.3	–	0.0	–	0.0	–	1.0	–	1.0	–	0.3	–	3.7		46.7	–	
9	4.5	–	5.3	–	1.3	–	0.0	–	0.0	–	0.0	–	0.0		0.0	–	
10	5.3	–	0.0	–	0.0	–	0.3	–	0.0	–	0.0	–	0.0		43.3	–	
11	2.7	–	0.0	–	0.0	–	0.0	–	0.0	–	0.0	–	0.0		0.3	–	
12	2.0	–	0.0	–	0.0	–	0.0	–	0.0	–	0.0	–	0.0		0.0	–	
13	–	9.5	–	0.3	–	0.3	–	0.8	–	0.0	–	2.5	–	0.0	–	0.0	
14	–	8.5	–	1.3	–	0.3	–	1.0	–	0.0	–	0.5	–	0.0	–	4.0	
15	–	7.8	–	0.0	–	0.0	–	0.3	–	1.0	–	1.0	–	1.3	–	0.0	
16	–	9.0	–	0.0	–	0.0	–	0.3	–	0.0	–	0.7	–	0.0	–	1.3	
17	–	9.0	–	1.7	–	0.0	–	2.0	–	1.7	–	3.0	–	2.0	–	4.0	
18	–	5.8	–	0.0	–	0.0	–	0.0	–	0.0	–	0.0	–	0.0	–	4.0	
19	–	6.3	–	0.0	–	0.0	–	0.0	–	0.0	–	0.0	–	0.0	–	5.0	
20	–	6.0	–	0.0	–	1.7	–	0.0	–	0.0	–	0.0	–	0.0	–	83.0	
21	–	5.3	–	1.3	–	0.3	–	0.3	–	0.7	–	0.3	–	0.7	–	7.0	
22	–	5.0	–	0.0	–	1.7	–	0.0	–	0.0	–	0.0	–	0.0	–	1.0	
23	–	3.7	–	0.0	–	0.0	–	0.7	–	0.7	–	0.7	–	0.0	–	0.0	
24	–	1.2	–	0.2	–	0.2	–	0.0	–	0.0	–	0.6	–	0.0	–	60.4	

Teams A1–20 participated at FIFA World Cup 2002 and 2006, Teams B1–24 qualified only once.

LA, local anaesthetics.

More than 10% of the players taking NSAIDs were using at least two preparations prior to a match (2002: n = 125; 2006: n = 102); in some players, as many as five different preparations were taken. Diclofenac was the most frequently reported active pharmaceutical ingredient (2002: n = 536 (48.2%); 2006: n = 550 (55.2%)), followed by Ketoprofen (2002: 150 (13.5%); 2006: 84 (8.6%)). The use of COX-2 inhibitors decreased from 2002 to 2006 (191 (17.2%) versus 101 (10.3%); p<0.001). Acetylsalicylic acid was rarely reported (2002: 30 (2.7%); 2006: 19 (1.9%)).

Other analgesics represented 5.5% of all prescribed medicines.

### Medications for the respiratory tract

Substances acting primarily on the upper and lower respiratory tract were the second most frequently prescribed substances (2002: 7.2%; 2006: 13.8%), with antihistamines being the most common of these, accounting for 40.7% (n = 186), followed by β2-agonists (n = 72, 15.8%), antitussives (n = 63, 13.3%), inhaled corticosteroids (n = 54, 11.8%), α-sympathomimetic agents (n = 52, 11.4%) and others (n = 30, 6.6%).

During the two tournaments, 11 players were reported to be using β2-agonists and NSAIDs together on 23 occasions.

### Corticosteroids

Corticosteroids – accounting for 7.2% of all medications – were mainly injected (n = 112, 35.2%) and used as ointments (n = 92, 28.9%). Other medical indications, such as for ears and eyes (n = 55, 17.3%), respiratory tract (n = 54, 17.0%) and gastrointestinal problems (n = 5, 1.6%) were less frequent.

### Others

In nearly 80% cases (119 out of 150), gastric protectors such as proton pump inhibitors (2002: n = 30; 2006: n = 21), prostaglandins (2002: n = 54; 2006: n = 32) and others (2002: n = 13; 2006: n = 0) were reported in combination with non-steroidal anti-inflammatory drugs.

Injections of local anaesthetics were reported 72 times in 2002 and 76 times in 2006.

Of all reported psychotropic substances, 94.1% were narcotics (2002: n = 111 of 118 cases, including 34 non-benzoic agents; 2006: 64 of 68 cases, including 24 non-benzoic agents); only three players were reported to use antidepressants.

### Nutritional supplements

**Figure 2:**
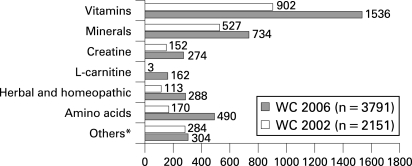
Reported use of nutritional supplements during FIFA World Cup 2002 and 2006. *Antioxidants, caffeine, CoQ10, essential fatty acids, taurine and others.

Significantly more nutritional supplements were prescribed in 2006 (1.28; 95% CI 1.25 to 1.33 supplements/player/match) than in 2002 (0.73; 95% CI 0.70 to 0.76; see fig 2): vitamins represented the majority (41.1%), followed by minerals (21.2%) and amino acids (11.1%).

Iron supplementation represented 11.8% of the prescribed minerals — substantially less than the mineral complexes (56.6%) and magnesium (20.6%). Of the amino acids, polyamino-acids were most often used (41.2%) followed by arginine (24.4%) and glutamine (22.0%). Ginseng supplementation represented more than half of the prescribed herbal and homeopathic substances (54.6%, 219 out of 401); preparations containing guarana (17.2%, 69) and arnica (11.5%, 46) were rarely used. Caffeine was reported as being used by two players in 2002 and four in 2006.

Some players were reported to take as many as ten different supplements prior to a game, and one team reported an average prescription of 7.4 nutritional supplements per player, per match during the FIFA World Cup 2006.

### Relation to team success and player exposure

For both tournaments, there was no significant correlation between the final ranking of the team and the mean number of medications taken per match, the mean intake of NSAIDs per match, or the mean intake of any substance per match (r between −0.15 and +0.06). Low but statistically significant correlations were found between a player’s average playing time per match and the mean number of medications taken per match (2002: r = +0.14, p<0.001; 2006: r = +0.16; p<0.001) and between the average playing time per match and mean intake of NSAIDs per match (2002: r = +0.14; p<0.001; 2006: r = +0.18; p<0.001).

## DISCUSSION

Sport in general has consistently been shown to have beneficial health effects, resulting in fewer medical consultations^17^ and a lower use of medication in physically active people.^18^

Top athletes, however, face a higher incidence of osteoarthritis and greater risk of injury than employees in most other occupations.^19^ Competing athletes also seem to have a higher intake of medication than non-competing athletes.^18^ ^20^

Mottram^21^ postulated that there are four types of medication use in professional athletes: legitimate therapeutic use, performance continuation (treatment of sports injuries), recreational/social use, and performance enhancement. Athletes trying to enhance their performance by taking pharmaceutical agents and large quantities of nutritional supplements are predominantly healthy athletes.^22^

Whilst the present study was able to document the prevalence of medication use in top athletes, it was not able to scrutinise the underlying motivations for, or the likely implications of, such use.

The present results show a widespread use of prescribed medicines in professional football, with 0.8 substances per player per match being reported. NSAIDs represented nearly 50 percent of all substances, with one in three players being prescribed NSAIDs prior to a match, one in ten taking two or more different preparations, and some taking as many as five different preparations.

No relationship was observed between team success and the amount or type of prescribed medication. However, players with a high average playing time were prescribed more medications and more NSAIDS per match than were substitutes.

A wide range was reported for nutritional supplement use (0–7.4 nutritional supplements per player, per match), with as many as ten different substances being taken prior to a match in some cases. Coaches have been found to have a greater influence than doctors and sports dieticians on the nutritional supplements taken by athletes;^12^ this could have biased the figures of intake reported by the team physicians.

Earlier studies have analysed the use of prescription medicines and nutritional supplements in national Olympic squads participating in winter and summer sports,^9^ ^23^ in athletes during the Olympic Games (OG) (information gained from doping controls)^10^ and in professional soccer players during one season.^13^ In the latter study, data were collected from personal interviews with the athletes themselves.^13^ This differs from the method employed in the present study, in which information was obtained solely from the team physicians; further – but similarly to the investigation conducted by Corrigan *et al*^10^ – in the present study, information was collected for each participating player, prior to every match.

Compared with the information acquired during the doping controls at the Olympic Games in Sydney,^10^ significantly more football players did not use any substance (22% in Sydney vs. 38% here); however, more were reported to use NSAIDs. Similar results to the present study were found in Finnish athletes participating in the Winter Olympic Games.^23^ In professional football players in Italy, however, 86% were reported to be current NSAIDs users.^13^

As reported previously,^10^ antiasthmatic medication is rarely prescribed in international football. In the present study, 2.2% of all players (ten players treated with β-2-agonists, 13 with inhaled corticosteroids and ten with combined therapy) were being treated for asthma; this compares with 5.2% (607 out of 10 672) during the OG 2000^4^ (p<0.001), 4.2% (445 out of 10 653) during the OG 2004^4^ (p<0.001) and 7.0% (31 out of 446) of all Finnish athletes competing at the Winter OG 2002(p<0.001).^23^

A total of 148 injections of local anaesthetic were reported, that is, nearly 1.2 injections per match. Similar incidences have been reported for rugby and for Australian rules football (1.7 and 1.4 injections per match, respectively).^24^

Although there are reports of a high prevalence of sustained adverse effects with NSAID use in athletes^23^ and alternative substances are well-known,^6^ ^25^ the indication for NSAIDs appears to have been broadened to almost any painful condition.^26^ The current literature does not provide any conclusive evidence to justify this high usage. On the one hand, the pain-relieving qualities of NSAIDs are uncontested;^5^ however, their influence on the healing process is controversial^5^ and their adverse effects in sport, for example in the context of dehydration and the kidney, are not fully understood.^7^ The success of therapy seems to depend on many injury-specific^21^ ^27^ and pharmacological variables.^5^ Animal studies have found potentially deleterious effects of NSAIDs on the healing process.^28^ Porucznik *et al* found a higher prevalence of suprascapular neuropathy in collegiate volleyball players who used NSAIDs than in athletes who did not.^29^ Paolini and Orchard^6^ discussed the issue of soft-tissue injuries and concluded that paracetamol had similar efficacy to NSAIDs in soft-tissue injury but had a lower side-effect profile. The authors concluded that paracetamol is the analgesic of choice for most soft tissue injury.^6^

The NSAID guidelines from the National Health Service^16^ recommend:

- Lowest possible dose and for shortest possible period.
- One preparation at a time.
- Prudent application in asthmatic patients.
- Avoid long-term use.
- Lowering gastrointestinal adverse effects by paracetamol with or without codeine.
- Use gastroprotective agents and or/COX-2 inhibitors in patients at high risk of gastrointestinal bleeding for whom NSAID therapy is necessary.

These therapeutic recommendations have not yet been adopted in international football. In the present study, more than one in ten players taking NSAIDs were using at least two preparations, NSAIDs were used 8.5 times more frequently than other analgesics, 10% of all players used NSAIDs prior to each of their matches, and 11 players were using antiasthmatic medication and NSAIDs at the same time. These findings are not unique to football, however.^10^ ^23^ ^29^

The substantial variation in the participating teams’ reported medication use – especially with respect to NSAIDs – highlights the difference in therapeutic concepts that currently exists in sports medicine; the majority are most likely based on individual empirical evidence or experience rather than on any firm evidence base.

In general, nutritional supplementation is not considered necessary, for either nutritional or immunological reasons, in athletes with an “adequate” diet.^30^ Additionally, there is not only the possibility of contamination,^31^ but also the potential for detrimental effects if different substances are misleadingly taken in excess.^30^ When competing at top level, therefore, professional advice on the quality and quantity of supplementation is essential.

## CONCLUSION

The high intake of non-steroidal anti-inflammatory drugs reported during consecutive FIFA World Cups is difficult to interpret, since the differentiation between indication, wrong application and misuse of prescribed medication is difficult to ascertain. Since the current literature does not support the extensive use of NSAIDs as a painkilling agent, dialogue with the treating physicians should be initiated, to provide a better understanding of this issue. Furthermore, prospective clinical studies should be carried out, especially within the context of analgesic use, in order to develop better, evidence-based recommendations for the treatment of sports injuries.

**What is already known on this topic**

In the literature to date, the main focus of drug use in professional football players has been on doping substances. However, the extent of the use of “legally” prescribed medication—which may have just as detrimental an effect on the player’s health—is largely unknown in professional football.

**What this study adds**

Professional football players—mostly healthy athletes—consume a considerable quantity of medication, especially non-steroidal anti-inflammatory drugs, prior to competition. These alarming results should initiate debate on the prescription and indications for medication in sport.
